# First report on *Babesia* cf. *microti* infection of red foxes (*Vulpes vulpes*) from Hungary

**DOI:** 10.1186/s13071-015-0660-5

**Published:** 2015-01-27

**Authors:** Róbert Farkas, Nóra Takács, Ákos Hornyák, Yaarit Nachum-Biala, Sándor Hornok, Gad Baneth

**Affiliations:** Department of Parasitology and Zoology, Faculty of Veterinary Science, Szent István University, Budapest, Hungary; Veterinary Diagnostic Directorate, National Food Chain Safety Office, Budapest, Hungary; School of Veterinary Medicine, Hebrew University of Jerusalem, Jerusalem, Israel

**Keywords:** Red fox, *Vulpes vulpes*, *Theileria annae*, *Babesia* cf. *microti*, Hungary

## Abstract

**Background:**

To date, only one report of a small *Babesia* infection based on microscopic observation which caused babesiosis in two dogs in Hungary has been published. Babesiosis due to *Babesia canis* - which is endemic in the local dogs - has only been detected in captive grey wolves. No information is available on babesial/theilerial infections in red foxes in Hungary. The aim of the study was to screen red foxes in Hungary for babesial parasites by PCR and to compare their partial 18S rRNA gene sequences to those parasites of domestic dogs and wild canids from other countries.

**Methods:**

Blood samples of 404 red foxes originating from 316 locations representing all 19 Hungarian counties were screened in Hungary for babesial parasites by PCR and the partial 18S rRNA gene sequences were compared to those parasites of domestic dogs and wild canids from other countries.

**Results:**

Altogether 81 red foxes out of 404 (20.0%; 95% CI: 16.4–24.2%) shot in 74 locations and in 17 of the 19 Hungarian counties were found to be infected with *Babesia* cf*. microti* by PCR.

**Conclusions:**

This is the first report to demonstrate the occurrence of *Babesia* cf. *microti* in Hungary, and its widespread presence in the fox population throughout the country. Further studies are needed to identify the tick species involved in its transmission, and whether other mechanisms of transmission are involved in its spread in fox populations.

## Background

Tick-borne piroplasmoses, caused by several intraerythrocytic apicomplexan protozoa, occur in a wide variety of vertebrates worldwide. Morphologically distinct large and small babesiae cause canine babesiosis in many parts of the world [1]. For many decades it was assumed that *Babesia canis* and *Babesia gibsoni* cause disease in dogs, and all small babesiae found in dogs were considered as *B. gibsoni* [2]. The genetic analyses of small babesiae of dogs have revealed that *B. gibsoni*, *Babesia conradae,* and the *Babesia* cf. *microti* species are phylogenetically close to zoonotic *B. microti* of rodents [3-5]. Zahler et al. [6] were the first to describe *Babesia* cf. *microti* infection from a German dog which had clinical babesiosis characterized by lethargy, fever, and anaemia after arriving back from Spain, and they proposed the name *Theileria annae* for the piroplasm***.*** The *Babesia* cf. *microti* species is also referred to as the “Spanish dog isolate” [1,7]. *Babesia* cf. *microti* infection was reported to be hyperendemic in Galicia, northwestern Spain, and was detected in 157 local dogs belonging to different breeds, coming from both urban and rural areas [8]. Infection with *Babesia* cf. *microti* caused disease associated with severe haemolysis, intense regenerative haemolytic anaemia, thrombocytopenia and azotaemia related to renal failure which was the main cause of death implicated in Spanish dogs [9-11]. A few years later Yeagley et al. [7] first reported that this organism also occurred in a dog in North America when one out of 157 dogs was positive for the canine small *Babesia* ‘Spanish isolate’. *Babesia* cf. *microti* parasites have also been detected in dogs from Croatia [12] and Portugal [13].

With regard to fox infection, Criado-Fornelio et al. [4] reported that they detected *Babesia* cf. *microti* DNA in 5 red foxes when studying frozen DNA samples obtained from the spleen of 10 foxes captured in central Spain during 1997–1999. A BLAST search in GenBank® revealed 100% similarity of Spanish red fox (*Vulpes vulpes*) isolate sequence and *T. annae* found in dogs. Further studies confirmed that the occurrence of this small protozoan is not uncommon in foxes in Spain which seemed to be an important wild reservoir of this pathogen [14-16]. *Babesia* cf. *microti* or *T. annae* has also been detected in foxes from other European countries, such as Italy [17-19], Croatia [20] Portugal [21] and Germany [22] and Austria [23]. A genetically and morphologically similar parasite has also been identified in foxes from North America [24,25]. When partial 18S ribosomal ribonucleic acid and beta tubulin gene sequences of the North American parasites detected from red fox (*Vulpes vulpes*) and gray fox (*Urocyon cinereoargenteus*) samples from Canada and the USA were analysed they were nearly identical to those ones previously reported from American foxes and Spanish dogs [26]. The tick vector species which transmits *Babesia* cf. *microti* is currently unknown. Camacho et al. [27] hypothesized that the European hedgehog tick *Ixodes hexagonus* could be the main candidate vector based on the higher prevalence of this tick species on dogs infected with this small *Babesia*, however this hasn't been proven.

Canine babesiosis caused by the large *Babesia* species, *B. canis* is endemic in Hungary [28,29]. To date, only one report of a small *Babesia* infection based on microscopic observation which caused babesiosis in two dogs in Hungary has been published [30]. Among wild canids, babesiosis due to *B. canis* has only been detected in captive grey wolves (*Canis lupus*) [31]. To our knowledge, no information is available on babesial/theilerial infections in red foxes in Hungary. Therefore, the purpose of this study was to screen red foxes in Hungary for babesial parasites by PCR and to compare their partial 18S rRNA gene sequences to those parasites of domestic dogs and wild canids from other countries.

## Methods

### Collection of samples

**Figure 1:**
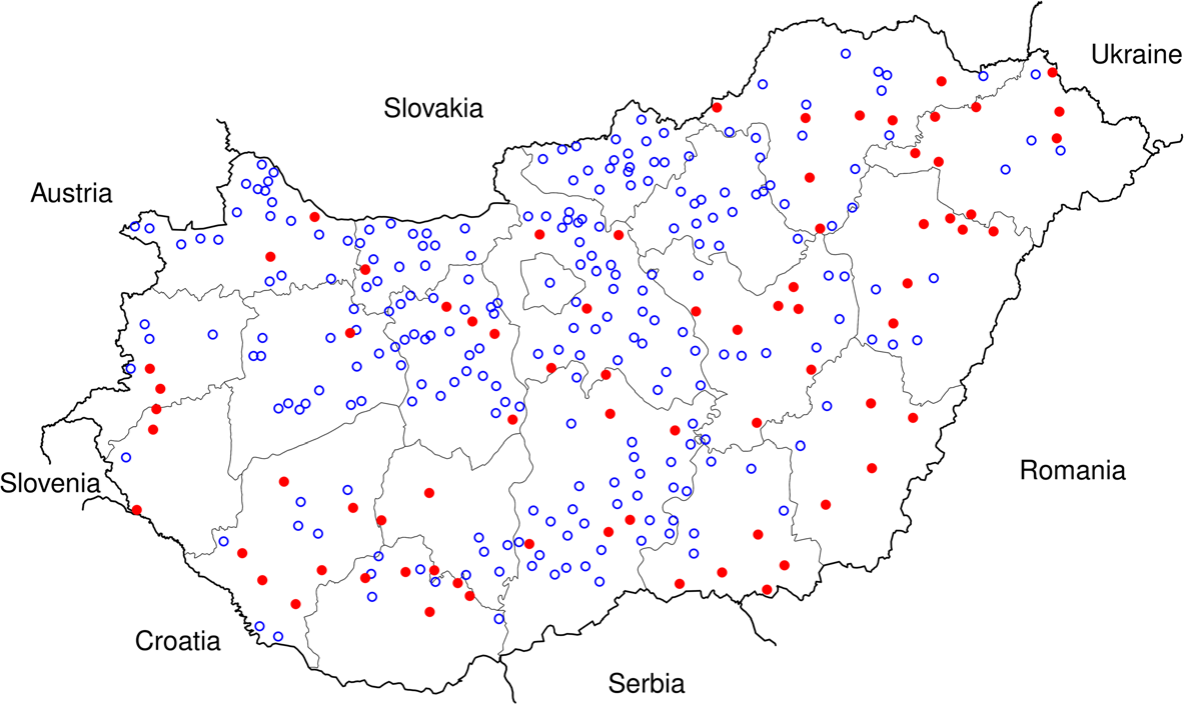
**The sampling sites of red foxes and where (red dots) foxes were found to be infected with** ***Babesia*** **cf.** ***microti*** **parasites in Hungary.**

Blood samples were collected from 404 red foxes originating from 316 locations representing all 19 Hungarian counties between June and October 2011 (Figure 1). The foxes were shot and the carcasses were sent to the Veterinary Diagnostic Directorate, National Food Chain Safety Office, Budapest, as part of a control program on oral immunization of foxes against rabies. After opening the thoracic cavity of foxes, blood samples were obtained via cardiac puncture from the right atrium or chest cavity and were then frozen at −20°C until further processing. Gender of foxes was not recorded.

### Ethical approval

The study was carried out in compliance with the ethical guidelines for study of wildlife animals in Hungary, and in agreement with the national animal welfare regulations (28/1998), and was approved by the Ethics Committee of the Faculty of Veterinary Science (SZIU).

### DNA isolation, amplification and sequencing

DNA was extracted from each blood sample using the QIAamp DNA Mini Kit (QIAgen GmbH., Hilden, Germany) following the ‘‘Blood and body fluid” protocol instructions by the manufacturer. A conventional single step PCR was used to amplify a 487 bp long fragment of the 18S rRNA gene of piroplasms with primers BJ1 [5’- GTC TTG TAA TTG GAA TGA TGG-3’] and BN2 [5’- TAG TTT ATG GTT AGG ACT ACG-3’] [32]. Reaction mix contained 15.9 μl sterile deionized water, 2.5 μl of 10× concentration of CoralLoad Buffer (15 mM MgCl_2_ included), 0.5 μl 10 mM dNTP, 0.5 μl of each primer (50 μM) and 0.1 μl (5 U/μl) of HotStarTaq Plus DNA Polymerase in a final volume of 25 μl containing 5 μl DNA. Amplification was performed with a BIOER GenePro BIOER TC-E-BD device (Bioer, Hangzhou, PR China). Initial denaturation at 95°C for 10 minutes was followed by 40 cycles of denaturation at 95°C for 30 s, annealing at 54°C for 30 s and elongation at 72°C for 40 s. The thermal program was finished with 5 minutes of final elongation at 72°C. Selected PCR products were purified and sequenced by Biomi Inc. (Gödöllő, Hungary).

### Phylogenetic analysis

A phylogenetic analysis, which included DNA sequences from foxes from this study, was carried out to compare these sequences to other *Babesia* spp. sequences deposited in GenBank®. *Hepatozoon canis* sequences were used as an outgroup [33]. Sequences were analyzed using the MEGA version 6.06 (http://www.megasoftware.net) and a phylogenetic tree was constructed by the Maximum likelihood algorithms using the Tamura 3-Parameter model. Bootstrap replicates were performed to estimate the node reliability, and values were obtained from 500 randomly selected samples of the aligned sequence data.

### Statistical analysis

Confidence intervals (CI) for the prevalence rates were calculated at the level of 95%.

## Results

Altogether 81 red foxes out of 404 (20.0%; 95% CI: 16.4–24.2%) were found to be infected with piroplasms by PCR. The positive animals were shot in 74 locations and in 17 of the 19 Hungarian counties except for Nógrád and Heves located in the northern part of the country close to Slovakia (Figure 1). When the 18 s RNA gene fragments from 30 positive foxes from 17 counties were sequenced and compared by BLAST with 18S rRNA sequences of *Babesia* and *Theileria* spp. available in GenBank, only *Babesia* cf. *microti* was detected. BLAST search revealed that all the submitted representative sequences obtained from 14 Hungarian foxes to GenBank (GenBank accession no. KM232509-22) were 100% identical to *B. microti*-like from a fox in Croatia (GenBank accession no. HM212628.1) as well as to other *B. microti*-like sequences from Italy (GenBank accession no. KF773740.1) and from *I. hexagonus* ticks found on a German red fox (GenBank accession no. JX679168.1).

**Figure 2:**
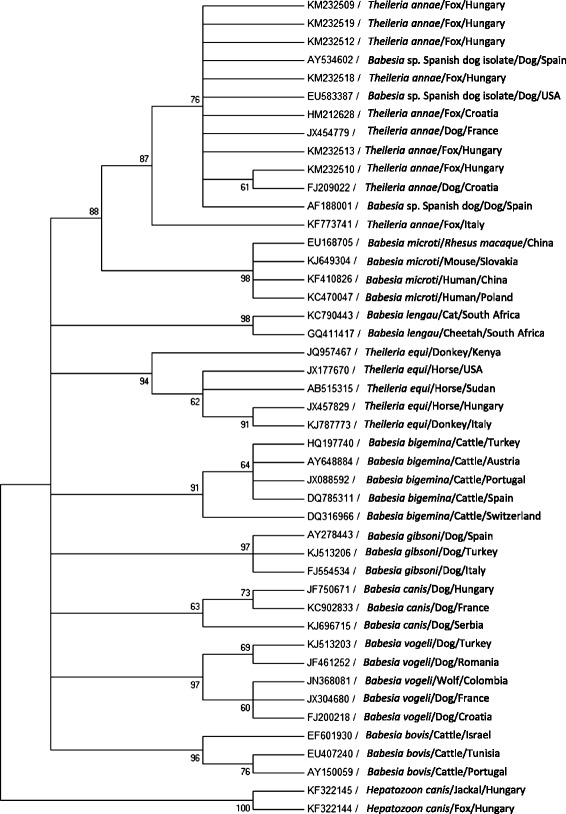
**Maximum likelihood 18S tree** ***;*** **A Maximum likelihood tree phylogram comparing 392 bp 18S DNA** ***Babesia*** **cf.** ***microti*** **(** ***Theileria annae*** **) sequences from Hungary included in this study to other** ***Babesia*** **and** ***Theileria*** **spp. sequences deposited in GenBank**®***.*** *Hepatozoon canis* sequences were used as an outgroup. The GenBank accession numbers, animal host species and country of origin from which the sequences were derived are included for each sequence. All six *T. annae* GenBank accesions from Hungary included in the phylogram are derived from foxes included in the current study.

A Maximum likelihood phylogenetic tree based on 392 bp from the 18S rRNA gene indicated that sequences from foxes tested in this study clustered together with other sequences of *Babesia* cf. *microti* or *T. annae* and separately from other piroplasmids including other *Babesia* spp. that infect canids (Figure 2).

## Discussion

The complete identity of *Babesia* cf. *microti* sequences detected from foxes in Hungary with *Babesia* cf. *microti* found in a fox in Croatia [20] allows us to suggest that *Babesia* cf. *microti* may have been introduced into Hungary from this neighbouring country. However, it cannot be excluded that this protozoan was transmitted from Hungary to Croatia by infected foxes. The occurrence of this blood parasite has been reported from another country neighbouring Hungary [23]; therefore, the parasite may have been introduced also from there. The fact that this protozoan parasite is widespread in the Hungarian fox population suggests that it was not recently introduced. The prevalence of *Babesia* cf. *microti* infection found in foxes in this study (20%) is higher than the prevalence of another tick-borne protozoan, *Hepatozoon canis*, recently described in red foxes in Hungary with a prevalence of 8%. Unlike *Babesia* cf. *microti*, *H. canis* is suspected to have been more recently introduced into Hungary, possibly from Croatia (33). The tick *I. hexagonus* suggested as the vector of *T. annae* in Spain [27] is known to be present in Central Europe [34], and also in Hungary [35]. According to the findings of an earlier Hungarian survey, *I. hexagonus* very rarely occurred among the 2500 ticks found on foxes [36], in contrast to the findings of a survey conducted in Germany where almost one-quarter of the 1953 ticks collected from foxes belonged to the species *I. hexagonus* [22]. In our opinion, although *I. hexagonus* may be the vector of this small piroplasm, involvement of other tick species in its transmission cannot be excluded. The latter possibility is supported by the fact that *Babesia* cf. *microti* occurs also in countries where *I. hexagonus* is either absent or uncommon [26]. According to German researchers [22], *I. ricinus* and *I. canisuga* may also act as a vector for *T. annae*, as ticks belonging to these species were often found on infected foxes. However, *Babesia* cf. *microti* DNA has been detected in *I. ricinus* as well as in *R. sanguineus* in Italy [37,38] and Spain [16] and *I. canisuga* in Germany [22]. The above-mentioned studies included ticks that have fed on foxes and therefore, being positive by PCR for the parasite might only signify that the blood meal contained its DNA, and not necessarily that the ticks are competent vectors in which the parasite can commence its life cycle. Therefore, further research is needed to clarify whether these tick species have vector competence for transmitting *Babesia* cf*. microti*. It is also possible that non-vectorial pathways of transmission may be responsible for the wide distribution of *Babesia* cf. *microti* in Hungary. According to Portuguese [13] and Swedish [39] researchers, this parasite may be capable of vertical spread and intrauterine transmission to the fetus, as its occurrence has been demonstrated in puppies free of tick infestation. It has also been suggested that, like *B. gibsoni* [40], *Babesia* cf. *microti* piroplasms can be transmitted among animals through bite wounds [7,26].

Further studies are needed to determine whether this parasite species can cause disease in foxes in the same way as it does in dogs. So far, there has been only a single report on clinical signs caused by *T. annae* in a fox [25]. In view of the widespread and common infection of foxes [22], we presume that this parasite is not pathogenic to foxes, and that foxes only act as its reservoir. Another question that remains to be answered is whether the golden jackal (*Canis aureus*), a wild canine species with a south to north range expansion in Hungary [41], can be a reservoir of this parasite. The parasite can probably be transmitted from foxes to dogs by ticks; however, currently no massive outbreaks such as those reported from Spain have occurred [8,10,11]. In Hungary, babesiosis caused by small babesiae has hitherto been demonstrated only in two dogs [30]; however, the species could not be identified by microscopic examination, as genetic analysis of the parasite was not possible. It has long been known that *B. canis,* a species causing severe disease in dogs, is present throughout Hungary [28,29]; therefore, the question arises whether or not the immune response and seropositivity of dogs to this species would prevent the development of clinical signs caused by *Babesia* cf. *microti* piroplasms*.* We have not had any knowledge whether detectable antibodies to this piroplasm species are produced in the foxes or in the affected dogs. It also requires explanation why *B. canis* did not occur in any of the several hundred foxes included in the study, when previous studies have demonstrated that the tick *D. reticulatus* transovarially transmitting this protozoon is a common ectoparasite of foxes [36]. Infection of red foxes with *B. canis* has been previously reported, but the parasite was identified only by microscopic examination [42]. During studies conducted in Croatia [20], *B. canis* was not detected in foxes even by molecular methods, although canine babesiosis caused by this blood parasite is also known to be common in that country [12]. Recently, Portuguese authors have reported, for the first time, the detection of *B. canis* infection by PCR in only one out of 91 red foxes examined [21]. In spite of the studies published on this parasite so far, our knowledge of its effect on the animal host's health is insufficient, and it is not known whether this parasite has strains of different virulence. According to the studies conducted to date, the host range of *Babesia* cf. *microti* extends beyond domestic and free-living carnivores, as this parasite has been detected also from other animal species including the horse [17], donkey [15], and cat [14]; however, its ability to induce disease in these latter species is unknown. In addition to the need to identify the tick species involved in transmitting the parasite, it remains to be determined whether ticks transmit this parasite only transstadially, as is the case with *B. microti* [43], or also transovarially, thus ensuring its survival and spread of in multiple tick generations.

## Conclusions

In summary, this is the first report to demonstrate the occurrence of *Babesia* cf. *microti* in Hungary, and its widespread presence in the fox population throughout the country. Further studies are needed to identify the tick species involved in its transmission, and the parasite’s mechanisms of transmission.
